# Adhesion of monocytes and endothelial cells isolated from the human aorta suppresses by miRNA-PEI particles

**DOI:** 10.1186/s12872-021-02203-2

**Published:** 2021-08-16

**Authors:** Adeleh Poursaleh, Farnaz Sadegh Beigee, Golnaz Esfandiari, Mohammad Najafi

**Affiliations:** 1 Biochemistry Department, Microbial Biotechnology Research Center, Iran University of Medical Sciences, Tehran, Iran; 2 Masih Daneshvari Hospital, Shahid Beheshti University of Medical Sciences, Tehran, Iran

**Keywords:** Endothelial cell, miR-125, miR-495, ICAM-1, ICAM-2, ITGB-2, VCAM-1

## Abstract

**Background:**

Knowledge of stenosis in coronary arteries requires an understanding of the cellular and molecular processes that occur throughout the leukocyte rolling process. In this study, the roles of miR-125a-5p and miR-495-3p were investigated on the adhesion of endothelial cells (ECs) isolated from the human aorta.

**Methods:**

Human primary endothelial cells were obtained from the aorta of people who had died of brain death. Whole blood was used to isolate the monocytes. The miR-125 and miR-495 were predicted and transfected into ECs using Poly Ethylene Imine (PEI). The expression levels of adhesion molecules and monocyte recruitment were identified by the RT-qPCR technique and Leukocyte-Endothelial Adhesion Assay kit, respectively.

**Results:**

The ICAM-1, ICAM-2 and VCAM-1 expression levels decreased significantly in the miR-495/PEI-transfected ECs (*P* < 0.05) while in the miR-125/PEI-transfected ECs only the ICAM-2 and ITGB-2 expression levels decreased significantly (*P* < 0.05) as compared to the miR-synthetic/PEI-transfected ECs. Furthermore, the monocyte adhesion was decreased in the miR-125 and miR-mix/PEI-transfected ECs as compared to the miR-synthetic/PEI-transfected ECs (*P* = 0.01 and *P* = 0.04, respectively).

**Conclusion:**

According to the findings, the efficient relations between miR-125 and adhesion molecules may be responsible for the inhibition of monocyte rolling.

## Introduction

Atherosclerosis is the leading cause of death from cardiovascular diseases (CVD) worldwide [1, 2]. In addition to the influence of lifestyle, several artery cellular dysfunctions are linked to the development of atherosclerosis [3]. The sub-endothelial macrophages, vascular smooth muscle cells (VSMCs), and endothelial cells (ECs) are the most important agents involved in the initiation and progression of atherosclerotic plaques in the heart arteries [4, 5]. Furthermore, the degree of cellular dysfunction is mediated by inflammatory events. The endothelial cells of arterial vessels trigger the leukocyte rolling process through the adhesion molecules. After the entrance of leukocytes mainly monocytes and T lymphocytes, immune-attractant and chemo-attractant reactions follow the progressive occurrences for the formation of atherosclerotic plaques in vessel sub-endothelial space causing the vessel micro-anatomical alterations and developing the extracellular matrix remodeling events [6]. The thrombotic problems during the progression of the atherosclerosis process may arise plaque ruptures led to vessel stenosis [7–9]. Many molecular and genetic studies have found that certain regulatory abnormalities in cellular signaling pathways lead to atherosclerosis. It is well known that miRNAs regulate the function of vascular smooth muscle cells (VSMCs) and modulate the inflammatory responses in vascular endothelial cells so that these events affect vessel stenosis and restenosis [10–13]. Moreover, the miRNAs have been discovered as potential therapeutic targets and clinical biomarkers in coronary artery diseases [14]. In VSMCs, miRNAs influence gene expression levels and control cellular signaling pathways [15–22]. A review of the roles of miRNAs in endothelial cell homeostasis has been published [23]. The miRNAs are also said to have many roles in the functional balance of vascular endothelial cells and circulating leucocytes via biological pathways and inflammatory responses [24].

According to the aforementioned descriptions, adhesion molecules facilitate leukocyte recruitment via the rolling process, which is a crucial phase in vascular stenosis and restenosis [25, 26]. Moreover, many studies also suggested that miRNAs influence the gene expression levels of adhesion molecules [27]. Based on miRNA-related databases, we projected miR-125a-5p and miR-495-3p and examined their impacts on ICAM1, ICAM2, VCAM1, and ITGB2 gene expression levels isolated from human aortic endothelial cells.

## Materials and methods

### Tissue sample

The normal aortic samples were collected postmortem from individuals with brain death by a specialist physician from the Organ Procurement Unit (Masih Daneshvari Hospital) (Subjects aged 21-54y were independently followed all steps; repeats, 3). The samples were submerged in saline/Amphotericin B (0.25 μg/ml)/Gentamicin (50 mg/ml)/Pen-Strep (%6, Gibco, Lot: 1697549) solution [28], and were safely transported into the central lab. The aorta's endothelial cells were quickly isolated on ice. The study has been authorized by the university's ethics committee (IR.IUMS.REC 1395.9274).

### Human aortic endothelial cell isolation

**Fig. 1:**
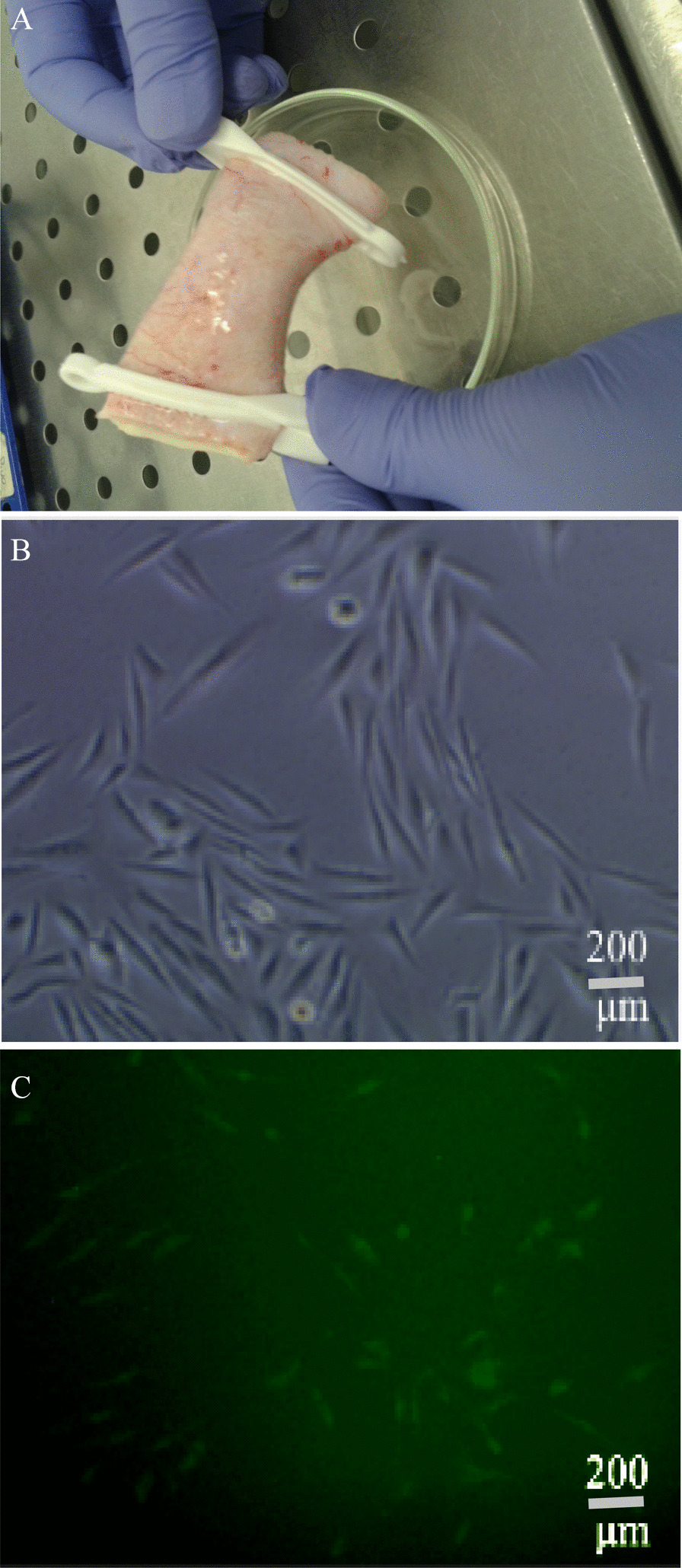
Isolation of endothelial cells from the human aorta. **a** The filled and clamped vessel with PBS buffer. **b** Endothelial cells. **c** FITC-miRNA/PEI-transfected complex to endothelial cells

A saline solution was used to cleanse the inner and outer surfaces of the aorta (length 8–10 cm). The two vessel sides were clamped after filling the aorta with PBS buffer (containing collagenase D 0.2%, Cat. No. C5138-100MG; Sigma Aldrich). Then, it was incubated for 30 min (37 °C, 5% CO2). The inner portion of the aorta was washed with free-serum Endothelial Cell Growth Medium MV (EGM-MV, PromoCell, C-22022) several times and the released endothelial cells (ECs) were collected in microtube (Fig. 1). The aorta's endothelial cell pellet was prepared by centrifugation technique (2500 rpm, 7 min), and was immediately seeded on the serum-EGM-MV medium (containing ECG complement, ECGS/H 0.004 ml/ml, hEGF 50 μg, HC 500 μg, FCS 5%, Pen-Strep 1% and amphotericin B (0.25 μg/ ml)). The endothelial cells (ECs) were trypsinized after 7 days. The cellular contents were centrifuged (2500 rpm, 5 min), and re-sedimented with cold PBS buffer (containing 10% FBS, Gibco™ 10091148). The cells were washed twice with cold PBS (5 min) and finally, the CD31^+^ cells were counted up to 56.7% using Flow Cytometry.

### LPS treatment

The aorta's endothelial cells isolated from aortic samples were quickly cultured on 6-well plates. The cellular groups were generally pretreated with 10 µg/ml LPS and were passaged three times depending on cell confluency (≈ 70%) (Cat. No.: L6529-1MG; Sigma Aldrich, Korea; Lipopolysaccharides from Escherichia coli 055:B5) for 6 h [29, 30].

### Gene and miRNA predictions

The genes implicated in the EC-leukocyte rolling process were obtained from network-based reactome (https://reactome.org) and text-based PubMed. STRING (https://string-db.org) was used to find the gene relationships based on the network edges. The genes were chosen through the network topology, high-evidence and high-express edges (Score > 0.9) obtained by Cytoscape software. Then, gene-related miRNAs were predicted from miRWalk server (http://zmf.umm.uni-heidelberg.de/apps/zmf/mirwalk2). The database reports were utilized as edge scores to hit gene-miRNA relationships.

### miRNA/PEI particles

The miRNA transfection was performed using Poly Ethylene Imine (PEI, Cat. No.: BCBS2233V). A solution of PEI (20 mg/ml DEPC water) was prepared with shaking at 37 °C. Also, a solution of each miRNA (including has-miR125a-5p TCCCTGAGACCCTTTAACCTGTGA, has-miR495-3p AAACAAACATGGTGCACTTCTT, miRsynthetic CCCGAGACCCAACTGGTCACC and miR-mix (containing equal amounts from miR125, miR495 and miRsynthetic) (100 pM) was prepared and incubated at room temperature for 20 min [31]. Then, 1 µl of each solution was added to 200 DEPC water and finally, the mixture was added into 800 µl culture medium. Based on the previous studies, the miRNA transfection rate was estimated up to 70% [16].

### EC transfection

After preheating the cells with LPS, the ECs were transfected with miRNA/PEI particles for 4 h. Finally, the cells were washed in PBS and were cultured in the serum-EGM-MV medium for 20 h.

### Monocyte isolation

Human monocytes were isolated using the RosetteSep kit (STEMCELL Technologies) [31]. Tetramer antibodies were added to the whole blood samples according to the producer’s procedure. The mixture was diluted with PBS buffer (volume ratio 1:1) and was centrifuged (3000 rpm, 20 min) with a ficoll gradient after incubation (37 °C, 20 min). Then, the monocytes were washed in a PBS buffer containing 2% fetal bovine serum.

### RNA extraction and cDNA synthesis

AccuPrep*®* Universal RNA Extraction Kit (Bioneer, Korea) was used to extract total RNA from PEI/miR-transfected ECs according to the manufacturer's instructions. Briefly, the endothelial cells were washed with cold PBS three times and, were harvested in lysis buffer (400 μl). After adding other buffers, ultimately the RNA sample was extracted and kept at − 80 °C. The cDNA synthesis was carried out using cDNA synthesis kit (Cat. No.: RR037A; Takara, Japan) according to the producer's instructions.

### Real-time qPCR method

The gene expression levels were measured using AB Applied Biosystems stepOne Real-Time PCR systems. The gene expression values were determined with SYBER Green PCR Kit (Cat. No.: RR820Q, Takara, Japan). In each reaction (15 µl), the forward and reverse primers (each 0.5 μM), cDNA (1 µl), and master mix (10 µl) were used to amplify the gene cDNA samples. The primers were designed using the Primer-BLAST tool. Furthermore, the beta-actin (ACTB) gene was applied as an internal reference (Table 1). The reaction cycles (n = 45) for all genes were followed after initial incubation at 94 °C for 2 min, 94 °C for 30 s, 67 °C (ICAM-1, ICAM -2 and ITGB-2), 63 °C (ACTB) and 55 °C for (VCAM-1) for 30 s.

**Table 1:** Primers

Primer	Forward-primer	Reverse-primer	Annealing temperature (°C)
ICAM-1	CAGTCAGTGTGACCGCAGAG	CGCCGGAAAGCTGTAGATGG	67
ICAM-2	GTCAGCGTGTACCAGCCTC	TCATTGCCACGGAACAGGAA	67
VCAM-1	TGTCAATGTTGCCCCCAGA	CACAGGATTTTCGGAGCAGG	55
ITGB-2	CTGTCGAACAACCCCGTGAA	CCACACACTCTCGGCTCTC	67
B-ACT	GCAAGCAGGAGTATGACGA	CAAACAAATAAAGCCATGCCAATC	63

### Immunofluorescence method

The aorta's endothelial cells (n = 100,000) were cultured for 72 h on 96-well plates. The cells were then pretreated with LPS (10 μg/ml, 6 h), washed with PBS (twice), and transfected by miRNA/EPI particles (4 h). Afterward, the cells were washed with PBS (twice) and were grown for 20 h. The ligand/LeukoTracker (No. 12101, Cell Biolabs) solution of CytoSelect ™ Leukocyte-Endothelial Adhesion Assay Kit (Cat. No.: CBA-210; Cell Biolabs; Denmark) was mixed with the isolated monocytes (as described in Seciiont 1.7) and was added to the cell culture for 2 h. Then, the cellular mixture was lysed (No. 10404, Cell Biolabs), stirred slowly, and finally the surface monocyte-EC interacted proteins were measured using a fluorescence plate reader (480 nm/520 nm).

### Statistical analysis

Data were analyzed statistically using a statistical software package (SPSS 24, Chicago). The gene expression levels were calculated using 2^−ΔΔCT^ formula. The Kolmogorov–Smirnov test was used to examine the data distribution. The differences between groups were evaluated by ANOVA and t-student tests. *P* values less than 0.05 were determined to be significant.

## Results

### miR-125a-5p and miR-495-3p are predicted for adhesion molecule genes

**Fig. 2:**
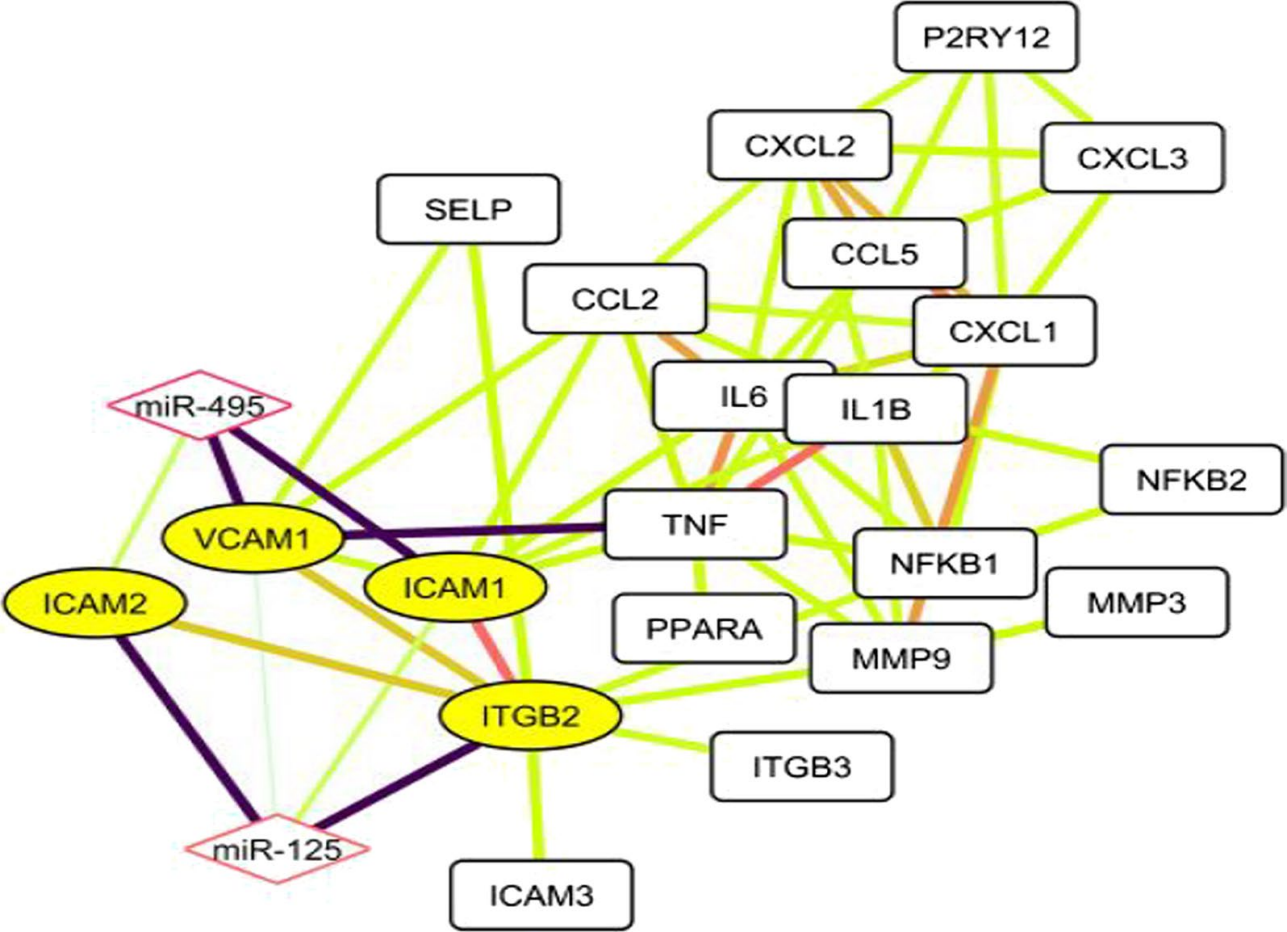
Gene and miRNA prediction. The gene network was drawn using Cytoscape based on reactome and PubMed searches. The high-evidence (dark edge) and high-express (yellow) nodes were selected from network. Then, miRNAs were predicted from databases

The high-score genes found using STRING and were subjects for the gene networking (node 23, edge 59). Based on the network topology, the genes of close together (yellow circular nodes, 4) searched to find high-report miRNAs. The miRNAs were added to the gene network so that the miR-125a-5p and miR-495-3p were chosen on the high-score edges (dark edge) (Fig. 2).

### miR-495 decreases ICAM-1 gene expression

**Fig. 3:**
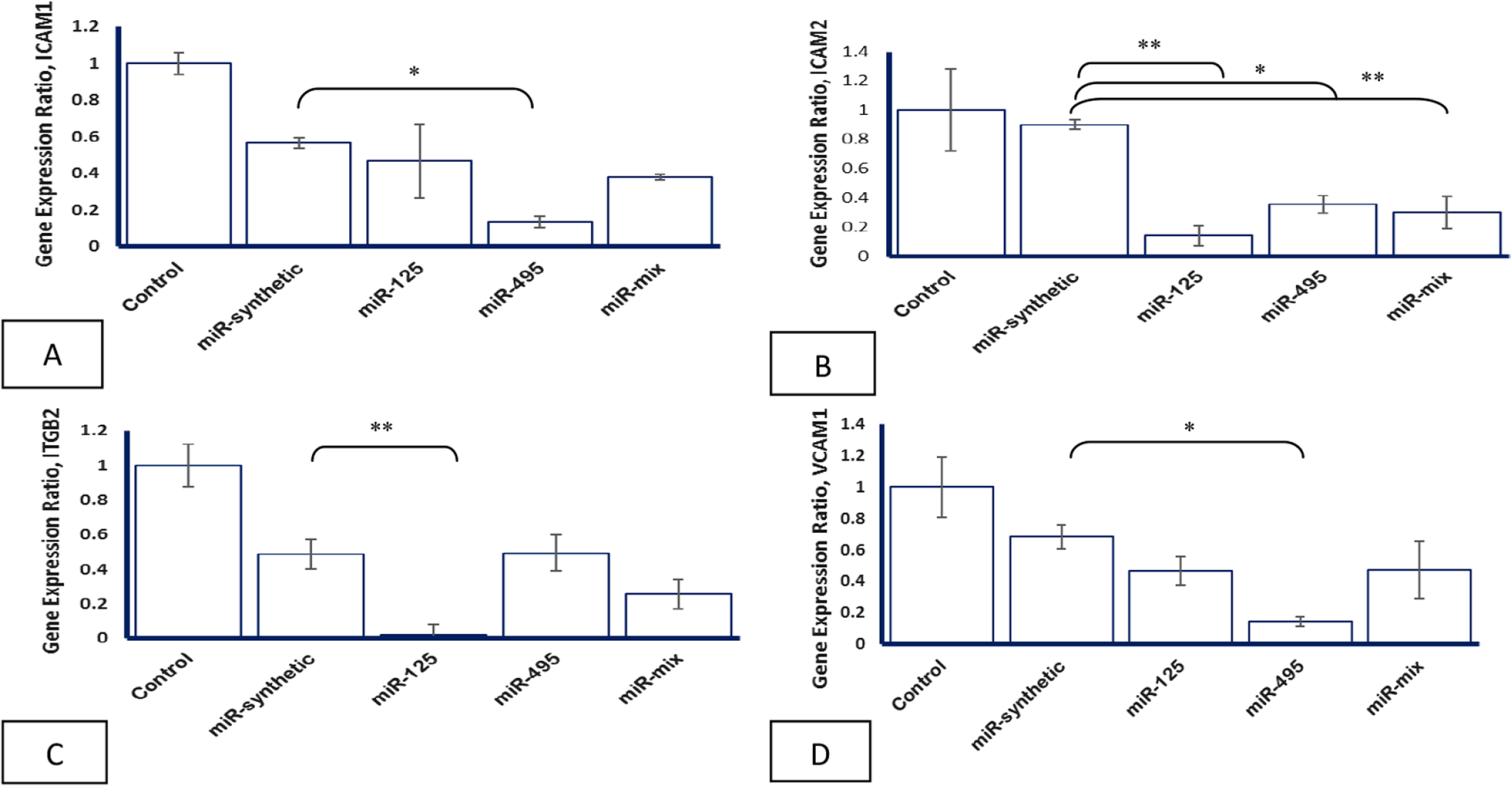
Effects of miRNA/PEI particles on the gene expression levels. **a** ICAM1. **b** ICAM2. **c** ITGB2. **d** VCAM1. The data are presented as means ± SD. *< 0.05, **< 0.005

The ICAM-1 gene expression level decreased significantly in the miR-495/PEI-transfected ECs as compared to miR-synthetic/PEI-transfected ECs (*p* 0.03). The results were not significant for miR-125 and miR-mix/PEI-transfected ECs (*p* 0.82 and *p* 0.39, respectively) (Fig. 3a).

### miR-125 and miR-495 decrease ICAM-2 gene expression

The results showed that the ICAM-2 gene expression levels decrease significantly in miR-125 and miR-495/PEI-transfected ECs as compared to miR-synthetic/PEI-transfected ECs about ten and three times, respectively (*p* 0.004 and *p* 0.012, respectively). Furthermore, the ICAM-2 gene expression decreased in the miR-mix/PEI-transfected ECs (*p* 0.009) (Fig. 3b).

### miR-125 decreases ITGB-2 gene expression

The ITGB-2 gene expression level decreased in miR-125/PEI-transfected ECs as compared to miR-synthetic/PEI-transfected ECs (*p* 0.001). However, the ITGB-2 gene expression level was not decreased significantly in the miR-495 and miR-mix/PEI-transfected ECs (*p* 0.9 and *p* 0.20, respectively) (Fig. 3c).

### miR-495 decreases VCAM-1 gene expression

The results showed that the VCAM-1 gene expression level decreases in miR-495/PEI- transfected ECs as compared to miR-synthetic/PEI-transfected ECs (*p* 0.02) (Fig. 3d).

### miR-125 and miR-mix decrease monocyte-endothelial cell adhesion

**Fig. 4:**
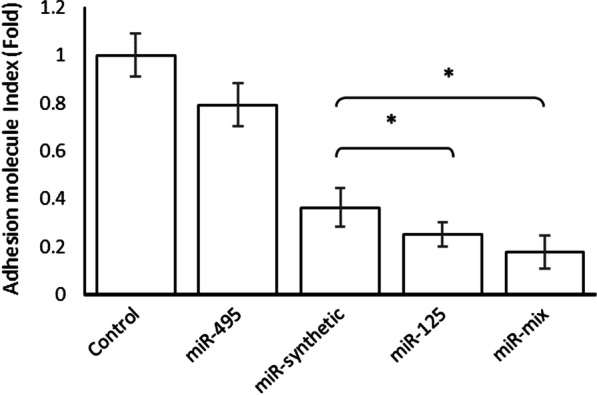
Adhesion molecule protein expression index. The adhesion rate between ECs and monocytes reduced for miR-125 and miR-mix as compared with miR-synthetic. *< 0.05

miR-495 had no significant effect on the endothelial-monocyte cell adhesion (*p* 0.86). The adhesion molecule protein values decreased significantly in the miR-125 and miR-mix/PEI- transfected ECs (*p* 0.04 and *p* 0.01, respectively) as compared to miR-synthetic/PEI-transfected ECs (Fig. 4).

## Discussion

The cellular responses of the vascular wall during the atherosclerosis process relate to the adhesion molecule functions, inflammatory events, lipid dysregulation, and extracellular remodeling dysfunction. Since the vessel restenosis is known as the most common problem following stenting thus studies on the cellular mechanisms involved in this process are of interest. It is well known that miRNAs change the extracellular and cellular proteomes via regulation of genes involved in the cellular signaling pathways. Since the polarization of macrophages and their roles in the progression of atherosclerosis plaques relate to monocyte rolling process thus the adhesion molecules presented on the surface of ECs are the most important miRNA-regulated gene targets that effectively affect the entrance of monocytes into subendothelial spaces [32–37]. There were the reports on circulating miRNAs as potential markers of the monocyte rolling and atherosclerosis events [38–41]. In this study, the effects of miR-495 and miR-125 on adhesion molecule genes (ICAM-1, ICAM-2, ITGB-2, and VCAM-1) were examined on the basis of prediction data in aortic endothelial cells.

Some studies suggested that miR-125 is expressed in ECs and VSMCs, and is closely linked to the activities of some cellular signaling pathways. In stroke-prone spontaneously hypertensive rats, miR-125 has been postulated as a key therapeutic component [42]. It was also reported as a cellular modulator [43], suppressing miR-125a-5p, which led to PDGF-induced VSMC proliferation and migration [44]. Furthermore, other studies have found that the miR-125a-5p modulates the PI3K/Akt/eNOS pathway, as well as apoptosis, inflammation, and mediates vasculoprotective effects in the endothelial cells [45–47]. Our study showed that the miR-125/PEI-transfected endothelial cells decrease the ICAM-2 and ITGB-2 expression levels. Furthermore, their ability for adhesion into monocytes was decreased, suggesting that adhesion molecule expression levels are primarily influenced by miR-125.

Furthermore, miR-495 has been shown to suppress tumor cell proliferation, migration, and invasion [48–51]. It is also important in the development of pluripotent stem cells into endothelial cells [52]. According to several studies, miR-495 reduces inflammatory events by inhibiting the inflammasome signaling pathway [53] and may act as a tumor suppressor by directly targeting PIK3R1 gene in endometrial cancer cells [54]. Moreover, the inhibition of miR-495 improved pulmonary vascular structural changes in mice [55]. The miR-495 also induced potent cardiomyocyte proliferation due to suppression of coactivator Cited 2 factor [56]. Furthermore, the miR-495 suppressed CCL2 expression and inhibited the EC proliferation and migration pathways [57–61]. Our study showed that the ICAM-1, ICAM-2, and VCAM-1 expression levels are suppressed in the miR-495/PEI-transfected aortic endothelial cells. The EC-monocyte cell adhesion levels also changed but not significantly in these cells. Based on the results of miR-mix/PEI-transfected aortic endothelial cells, this study suggested that the functional effect of a miRNA might be related cumulatively to other miRNAs in the cells. Furthermore, it is proposed that the cellular uptake and clearance of miRNA mixtures may be related to the numbers and sequences of delivered miRNAs in ex-vivo mRNA expression evaluations.

## Conclusion

The ICAM-1, ICAM-2, and VCAM-1 expression levels are related to miR-495. There were expression associations between the ICAM-2, ITGB-2, and miR-125. Furthermore, monocyte adherence to miRNA-transfected aortic endothelial cells confirmed the role of miR-125 and the cumulative effects of miR-495 on cellular adhesion, showing that miRNAs may inversely control leukocyte rolling process. The use of miRNAs may improve the effectiveness of drug-based approaches [62] in the treatment of vessel stenosis and re-stenosis. These components can develop the controlled drug delivery techniques in drug-eluting stents and drug-eluting balloons [63]. However, it was better to study the roles of miRNAs in vessel scaffolds to improve our understanding from diapedesis process.

## Data Availability

The data used and analyzed in this study are presented by the corresponding author on request.
